# Environmental Risk Factors Influence the Frequency of Coughing and Sneezing Episodes in Finisher Pigs on a Farm Free of Respiratory Disease

**DOI:** 10.3390/ani12080982

**Published:** 2022-04-11

**Authors:** Joana Pessoa, Jordi Camp Montoro, Telmo Pina Nunes, Tomas Norton, Conor McAloon, Edgar Garcia Manzanilla, Laura Boyle

**Affiliations:** 1 Pig Development Department, Teagasc Animal and Grassland Research and Innovation Centre, Fermoy, P61 C996 Cork, Ireland; jordi.montoro@teagasc.ie (J.C.M.); egmanzanilla@gmail.com (E.G.M.); laura.boyle@teagasc.ie (L.B.); 2 Section of Herd Health and Animal Husbandry, School of Veterinary Medicine, University College Dublin, Belfield, D04 V1W8 Dublin, Ireland; conor.mcaloon@ucd.ie; 3 M3-BIORES—Measure, Model & Manage Bioresponses, KU Leuven, B-3001 Leuven, Belgium; tomas.norton@kuleuven.be; 4 Animal Nutrition and Welfare Service, Department of Animal and Food Sciences, Universitat Autònoma de Barcelona, Bellaterra, 08193 Barcelona, Spain; 5 CIISA—Centre for Interdisciplinary Research in Animal Health, Faculty of Veterinary Medicine, University of Lisbon, 1300-477 Lisbon, Portugal; tnunes@fmv.ulisboa.pt

**Keywords:** ammonia, air quality, precision livestock farming, PLF, porcine respiratory disease complex

## Abstract

**Simple Summary:**

Inappropriate environmental conditions in pig buildings are detrimental for both pig and farm-staff health and welfare. With ongoing technological developments, a variety of sensor technology is available and can be used to measure environmental conditions such as air temperature, relative humidity, and ammonia and dust concentrations in real time. Moreover, a tool was recently developed to give farmers an objective assessment of pigs’ respiratory health by continuously measuring coughing in finisher pigs. This study assessed baseline levels of coughing on a farm free of respiratory disease, and aimed to identify relationships between environmental conditions and coughing frequency in pigs. Six replicates were conducted. Coughing levels in healthy pigs were overall low, and coughing frequency can be predicted by environmental conditions such as high ammonia concentrations and high ventilation rates. Results of this study can be used as guidelines to determine normal coughing levels in healthy pigs, and to calibrate the alarm systems of tools that measure coughing frequency, such as the cough monitor used in this study. The collection and amalgamation of data from a variety of sources related to health, welfare, and performance are important in order to improve the efficiency and sustainability of the pig industry.

**Abstract:**

This study aimed to assess baseline levels of coughing on a farm free of respiratory disease, and to identify relationships between environmental conditions and coughing frequency in finisher pigs. Six replicates were conducted (690 pigs in total). A cross-correlation analysis was performed and lags of the predictor variables were carried forward for multivariable regression analysis when significant and showing r > 0.25. Results show that coughing frequency was overall low. In the first replicate, coughing was best predicted by exposure to higher ammonia concentrations that occurred with a lag of 1, 7, and 15 days (*p* = 0.003, *p* = 0.001, and *p* < 0.001, respectively), while in the sixth replicate coughing frequency was best predicted by the exposure to lower relative humidity and higher ventilation rates with a lag of 7 and 15 days (*p* < 0.001 and *p* = 0.003, respectively). Ammonia concentrations varied according to ventilation rates recorded on the same day (r > −0.70). In conclusion, guidelines on coughing levels in healthy pigs and calibration of the alarm systems of tools that measure coughing frequency can be extrapolated from this study. Environmental risk factors are associated with the respiratory health of finisher pigs.

## 1. Introduction

Respiratory disease remains a major economic and health concern in the pig industry worldwide [1,2]. Although there are several primary and opportunistic pathogens involved in the Porcine Respiratory Disease Complex (PRDC) [3,4], inappropriate thermal and gaseous environments in pig buildings can exacerbate transmission and spread of these pathogens, triggering and/or increasing severity of clinical outbreaks [3,5]. Furthermore, unfavorable environmental conditions act as a stressor and may damage the pigs’ respiratory tract [3]. Therefore, they have a detrimental effect on pig health, welfare, and performance [6].

Ammonia (NH_3_) is the most common health threatening gas in animal buildings [7]. Like several respiratory pathogens, NH_3_ depresses ciliary activity and mucus flow [8], impairing the mucosal clearance system, thus predisposing the respiratory tract of pigs to infections [9]. Another air pollutant that contributes to low air quality in pig buildings is ‘dust’ or particulate matter (PM) [9]. PM is a mixture of suspended materials, made up primarily of feed particles, dried fecal material, bacteria, and bacterial toxins [10]. These particulates cause inflammation or irritation of the pigs’ respiratory epithelium [9].

Temperature and relative humidity can also influence pigs’ respiratory health by disrupting the normal respiratory and thermoregulatory behavior of the animals, while contributing to the survival of pathogens [5,6]. Environment-oriented data, specifically data on air temperature and relative humidity, are routinely collected and used to regulate ventilation systems in most intensive pig farms. Unfortunately, the potential of such data to add value to pig health management on farm is rarely exploited [11]. Measurements of NH_3_ concentrations on-farm largely rely on portable sensors that give intermittent and short-term readings [11,12]. However, Dräger^®^ launched an electrochemical sensor in 2016 that is capable of continuously monitoring NH_3_ concentrations without requiring recalibration.

With ongoing technological developments, a variety of Precision Livestock Farming (PLF) tools are available [13,14]. In recent years, research efforts developed a tool to help monitor and control respiratory disease on-farm by performing continuous and automated measurements of coughing through the analysis of sound collected within pig buildings using a microphone [15]. A recent study employing such technology identified the need to classify patterns of coughing according to environmental risk factors, and to verify the baseline coughing frequency in healthy pigs [16].

Combining animal- and environment-oriented sensor data for the purpose of strategic decision-making is a promising area of future research [11] and could help address the challenge of the respiratory disease syndromes in pigs [17]. Indeed, the collection and amalgamation of data from a variety of sources (potentially including sensors on-farm) related to biosecurity, health, welfare, and performance should be considered a major priority for the efficiency and sustainability of the pig industry [18]. 

Therefore, the objectives of this study were (1) to assess baseline levels of coughing on a respiratory disease-free farm, and (2) to assess the relationship between environmental conditions (ammonia, particulate matter, temperature, and relative humidity) and levels of coughing.

## 2. Materials and Methods

### 2.1. Animals and Experimental Design

This study took place at the Teagasc Pig Research Facility in Fermoy, Co., Cork, Ireland from March 2019 to January 2020. It received ethical approval from the Teagasc Animal Ethics Committee (Approval number: TAEC 204/2018).

The farm operates as a farrow-to-finish facility with a three-week farrowing batch system. Routine seroprofiles show this farm was negative for Porcine Reproductive and Respiratory Syndrome virus, Influenza A virus, *Mycoplasma hyopneumoniae*, and *Actinobacillus pleuropneumoniae*. Moreover, abnormal cases are sent for analysis after necropsy and are consistently negative for the above-mentioned pathogens.

Danish Duroc X (Large White × Landrace) pigs were housed in rooms with 10 pens with fully slatted concrete floors (2.4 × 4.2 m), containing a wet-dry feeder (MA37, Verba, Sint-Oedenrode, The Netherlands) and one nipple drinker. Water and pelleted feed were provided ad libitum. Temperature was controlled with a temperature-based mechanical ventilation system (Big Dutchman 135pro, Vechta, Germany). Space allowance and mixing procedures are described in [19]. Briefly, space allowance varied between 0.78 m^2^/pig and 0.96 m^2^/pig (with 13 and 10 pigs/pen, respectively). In each room, five pens were left as intact litters and the other five pens were mixed balancing by weight and sex.

The rooms were illuminated artificially from 07:00 until 18:00 h and pens were enriched with a 1.20 m larch wood post fixed on one of the walls without impairing on the available floor space.

Three production batches of pigs (690 pigs in total) were each housed in two rooms (rooms A and B; 115 pigs per room) from 11 weeks of age, thus encompassing six replicates. All pigs were identified with ear-tags such that they could be monitored until reaching the target slaughter weight of 110 kg (with 21 to 23 weeks of age).

All seasons within a year were covered. The first batch was reared from March to May (spring), the second from July to September (summer), and the third from October to January (autumn/winter).

### 2.2. Data Collection

Data on environmental parameters and respiratory health were collected resulting in several datasets originating from different sensors and manual assessments. Moreover, lung lesions were scored at slaughter.

#### 2.2.1. Environmental Data

Environmental sensors were used to record daily measurements (average, minimum, and maximum values) of temperature and relative humidity. Sensors were placed in the center of each room. Temperature sensors were placed at a height of approximately 1.5 m and the relative humidity sensors were placed at approximately 2 m. These sensors were part of the cough monitor (SoundTalks NV, Leuven, Belgium) system.

The ammonia sensors (Dräger Polytron C300 with DrägerSensor NH3-Al, Lübeck, Germany) used in this study were electrochemical sensors that perform continuous long-term ammonia measurements. One NH_3_ sensor was placed in each room, following manufacturer’s guidelines. It provided data points for ammonia concentrations every 30 s.

Particulate matter (PM_10_ only) concentrations were measured in each room using a hand-held laser particulate counter (PCE-PCO 1, PCE Instruments, Meschede, Germany). This procedure was carried out approximately five days a week between 8 am to 1 pm, every week from the time the pigs entered the house until they were removed for slaughter.

Data on ventilation rates (air exchange in m^3^ per hour) were recorded for the whole duration of this study (Big Dutchman 135pro and Big Farm Net computer system, Vechta, Germany).

#### 2.2.2. Respiratory Health Data

The cough monitors (SoundTalks NV, Leuven, Belgium) used in this study perform continuous and automated measurements of cough sounds, issuing a Respiratory Distress Index (RDI) that corresponds to the average number of coughs per pig per twenty-four hours. They also generate an automated warning, which is set according to a patented Statistical Process Control algorithm using the history and the variation of the RDI from a specific room.

One cough monitor was installed in each room, following the manufacturer’s guidelines. All data collected during this study were stored and accessed using the associated pig respiratory distress monitoring (RDM) software.

For each room, the number of coughs and sneezes were counted over a 5-min period approximately five days a week, until pigs reached the target slaughter weight.

All assessments were performed following the Welfare Quality Protocol^®^ [20] guidelines. The average coughing frequency (CF) was estimated as:

$$\mathrm{CF}=\frac{\sum \mathrm{Number}\;\mathrm{of}\;\mathrm{coughs}\;\mathrm{in}\;a\;\mathrm{Room}}{\mathrm{number}\;\mathrm{of}\;\mathrm{examined}\;\mathrm{pigs}\;\left(n\right)*\mathrm{total}\;\mathrm{time}\;\mathrm{of}\;\mathrm{observation}\;\left(\min.\right)}$$

Sneezing frequency (SF) was estimated using the same formula. These data were collected at the same time as particulate matter concentrations were recorded.

#### 2.2.3. Slaughterhouse Data

Pigs were sent to the slaughterhouse when they reached 110 kg body weight (3 loads per batch with a one-week interval). A slap number linked to each pig’s ear-tag number was used, thus allowing us to match the carcass and offal to the corresponding room. All pluck examinations were carried out by the same trained veterinarian. 

Lung lesion scoring followed the same protocol reported in [16]. In summary, for each pig, individual lung lobes were examined for pneumonia lesions according to the method developed by Madec and Derrien [21]. The scores were 0 (no pneumonia) to 4 (76–100% of the lung lobe affected). Pleurisy was scored on the dorsocaudal lobes using a modified version of the Slaughterhouse Pleurisy Evaluation System [22]. The scores were 0 (no pleurisy), 2 (focal lesions in one lobe), 3 (bilateral adhesions or monolateral adhesions affecting more than 1/3 of the diaphragmatic lobe), and 4 (extensive lesions affecting more than 1/3 of both diaphragmatic lobes). Cranial pleurisy (CP) and pericarditis were recorded as absent (0) or present (1).

Individual cold carcass weights were also recorded.

### 2.3. Statistical Analysis

R version 4.0.2 (R Core Team, Vienna, Austria) was used for the statistical analyses [23].

Descriptive statistics are presented for all variables assessed.

#### 2.3.1. Sensor-Based Data

For all variables (temperature, relative humidity, ammonia concentrations, ventilation rates and the respiratory distress index), mean, standard deviation, median, minimum, and maximum values were calculated for rooms A and B in each batch.

Daily averages of NH_3_ concentrations and of ventilation rates were used.

To assess the relationship between the time series corresponding to temperature, relative humidity, NH_3_ concentrations, ventilation rates (the predictors), and the RDI (response variable), variables were first tested for stationarity using the Dickey-Fuller test. If non-stationary, differencing was applied.

Because respiratory health can depend on the exposure to the currents day’s environmental conditions (lag 0), but also on exposure on previous days (negative lags), a cross-correlation analyses was carried out in order to gain insight into the relationship between these variables, and to identify lags that may be useful predictors of the RDI. For biological reasoning, only negative lags and lag 0 were considered. Lags of the predictor variables were carried forward for multivariable regression analysis when significant and showing r > 0.25.

Models were constructed using a backward stepwise elimination based on the Akaike information criterion (AIC). Autocorrelation of residuals was assessed using the Breusch-Godfrey test. Only models where no autocorrelation of residuals was detected are presented. Bonferroni correction was applied to correct for multiple testing.

A cross correlation analyses was also carried out to assess the relationship between ventilation rates and NH_3_ concentrations.

#### 2.3.2. Manually Collected Data

Because these data were not continuous time series, a different statistical approach was taken. Spearman’s rank correlations were performed to examine the associations between particulate matter, NH_3_ concentrations, temperature, relative humidity, ventilation rates, and coughing and sneezing frequencies.

Day was used as the experimental unit. For sensor-based data, only the days when manual assessments took place were included (to avoid missing data). Alpha level for significance and tendency were 0.05 and 0.1, respectively.

## 3. Results

The duration of each finisher period was on average 78 (±1) days. Lung lesions were scored on 506 pairs of lungs (84 ± 22 pairs of lungs per replicate). In general, no gross pathology was observed in the lungs. However, the prevalence of pericarditis in pigs reared in the first batch in room A was 10%; moreover, 3 ± 2% of the trial pigs presented liver milk spots.

Table 1 shows descriptive results for all sensor-based variables.

**Figure 1:**
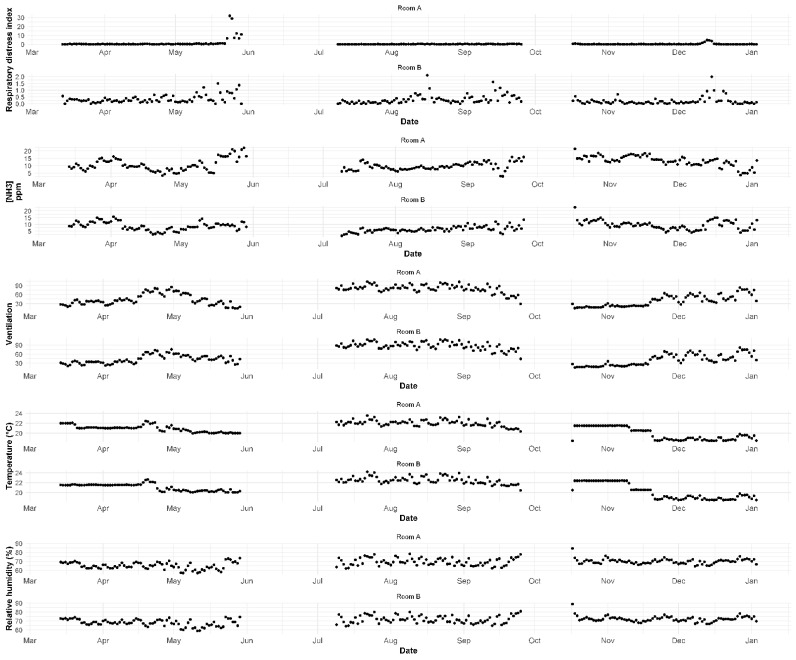
Daily averages of the respiratory distress index, three environmental parameters, and ventilation flow (air exchange in m^3^/h).

Overall, the respiratory distress index remained low throughout the six replicates assessed, with the exception of room A during the 1st batch where the RDI reached 31.9 at the end of the finisher stage (Figure 1).

Daily variation for all sensor-based variables can be seen in Figure 1. In general, higher daily values of NH_3_ concentrations were recorded in autumn/winter (3rd batch), followed closely by spring (1st batch). The inverse occurred for ventilation rates, which were higher during summer (2nd batch).

**Figure 2:**
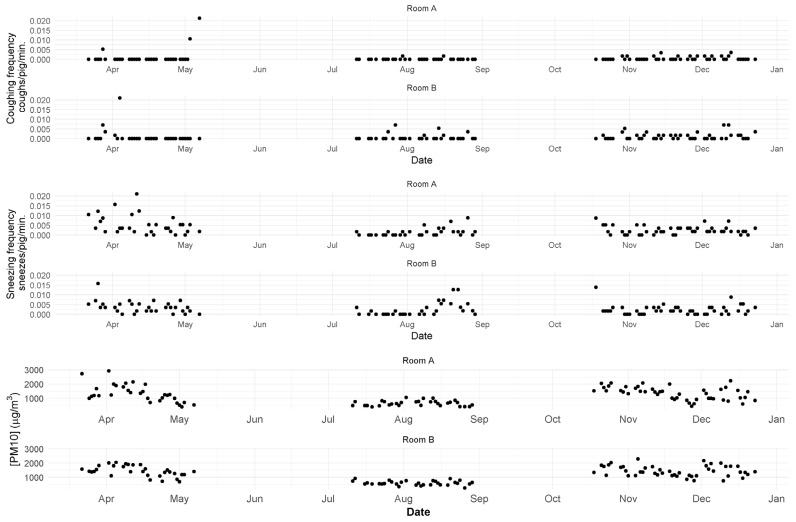
Intermittent measurements of the concentration of Particulate Matter (PM10) and coughing and sneezing frequencies assessed manually.

Table 2 shows descriptive results for all manually assessed variables. Overall, coughing and sneezing frequencies were low throughout all batches in the two replicates assessed, although CF was higher in room A during the 1st batch, when it increased by the end of the finisher stage, as did the corresponding RDI values (Figure 1 and Figure 2). Values of particulate matter were consistently low during the summer (Figure 2).

Results on the cross-correlation analysis between environmental conditions and the RDI are shown in Supplementary Material Figure S1.

The multivariable models fitted for the RDI in room A during the 1st batch and in room B during the 3rd batch are presented in Table 3 and were able to explain 47 and 39% of variability, respectively. In room A during the 1st batch the RDI was best predicted by exposure to higher ammonia concentrations that occurred with a lag of 1, 7, and 15 days (*p* = 0.003, *p* = 0.001, and *p* < 0.001, respectively), while in room B during the 3rd batch the RDI was best predicted by the exposure to lower relative humidity and higher ventilation rates with a lag of 7 and 15 days, respectively (*p* < 0.001 and *p* = 0.003, respectively).

**Figure 3:**
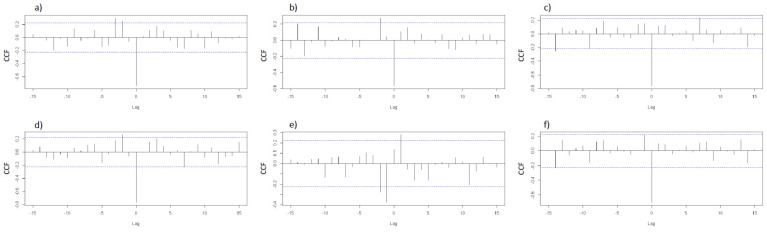
Cross-correlation function for ventilation rates and NH_3_ concentrations. Graphs (**a**–**c**) correspond to the first, second, and third batches of room A, respectively. Graphs (**d**–**f**) correspond to the first, second, and third batches of room B, respectively. Blue lines indicate the significance threshold. CCF: Cross-correlation function. Blue lines indicate statistically significant associations.

Regarding the cross-correlation analysis assessing the relationship between ventilation rates and NH_3_ concentrations, Figure 3 shows strong (r > −0.70) negative associations on lag 0, meaning that NH_3_ concentrations varied according to the ventilation flow recorded on the same day. However, during the 2nd batch, this association was lower (in room A) or not present (in room B).

**Figure 4:**
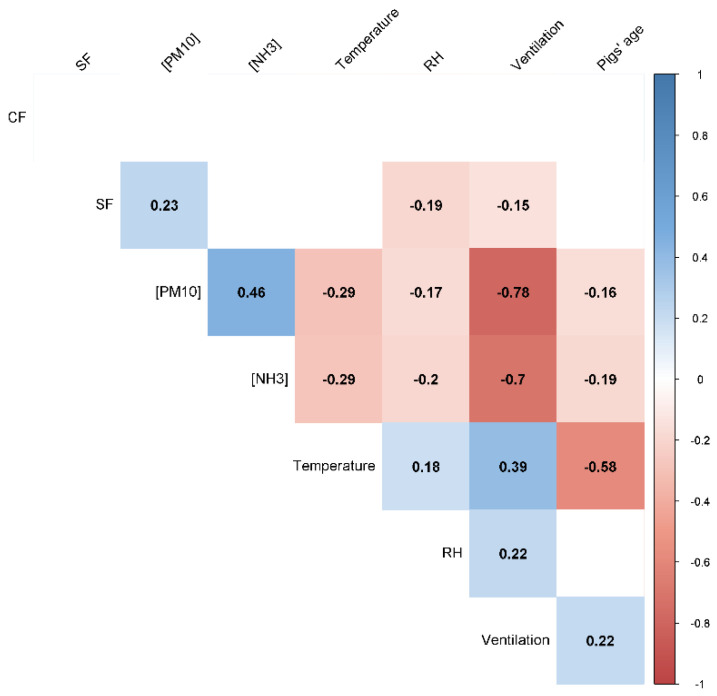
Spearman rank correlations between coughing and sneezing (CF and SF, respectively), concentrations of ammonia and particulate matter ((NH_3_) and (PM_10_), respectively), temperature, relative humidity (RH) and ventilation flow. Blank cells correspond to non-significant results (*p* > 0.050).

The findings from the univariable analysis of environmental conditions and coughing and sneezing frequencies assessed manually are presented in Figure 4.

There were no correlations between CF and environmental conditions. SF had a weak positive association with PM (*p* < 0.001), whereas PM was negatively correlated with ventilation flow (*p* < 0.001).

## 4. Discussion

In the current study, we aimed to explore how coughing frequency varied throughout the finisher stage on a farm free of respiratory disease, and to assess the relationship between environmental conditions within pig houses and coughing levels.

The findings of our study show that the frequency of coughing measured both by the cough monitor and manually were mostly low. Several studies measured the RDI in farms with a high prevalence of lung lesions [15,16] and/or in farms positive for different pathogens associated with the Porcine Respiratory Disease Complex [16,24]. When compared to our study, others generally reported higher values of the RDI, where it reached ≈ 10 [15,16] and ≈23 [24]. However, these studies also report low coughing levels throughout the finisher stage. Indeed, Polson et al. [24] associated an increase in coughing with positivity to Influenza A virus. A recent randomized control trial where pigs were inoculated with *Mycoplasma hyopneumoniae* (9 pigs per group with different ratios of inoculated pigs; control group: *n* = 3 pigs) reported RDI varying from 0.01 to 0.17 [25]. Higher coughing frequencies were reported in the groups where more inoculated pigs were present. However, the authors suggest that these low RDI values may be the result of the small number of pigs in each group, since the cough monitors were designed for larger pig populations [25].

Although more research is needed to understand how different coughing patterns associate with different pathogens, our study indicates that RDI values between 0–4 can be considered normal for intensive, indoor commercial farms.

However, we also recorded RDI values of ≈ 32 at the end of the finisher stage in one batch (first batch in room A). When exploring the relationship between environmental risk factors and coughing frequency, our model was able to explain 47% of the variability. Interestingly, in this case, the RDI was best predicted by the exposure to higher concentrations of ammonia occurring with a lag of 1, 7, and 15 days. In Figure 1, we can see that, by the end of finisher stage, a steep increase in NH_3_ precedes an increase in the RDI. To our knowledge, this is the first study evaluating the relationship between continuous measurements of coughing frequency and NH_3_ concentrations. However, in 1969, Stombaugh et al. [8] reported that at 100 and 150 ppm, pigs coughed three times more than those exposed to lower ammonia concentrations (10 and 50 ppm). Still, several other studies showed that (1) ammonia concentrations over 35 ppm induce inflammatory reactions in the respiratory mucosa of animals ([26] cited in [17]); (2) pleurisy is positively correlated with NH_3_ concentrations above 25 ppm [27]; (3) pigs exposed to NH_3_ concentrations varying from 0.6–37 ppm show small pathological changes in their respiratory tract [28]; and (4) that pigs show a preference for clean air when compared to air with varying NH_3_ concentrations [29,30,31]. Moreover, high concentrations of NH_3_ are relevant not only to animal health and welfare, but also to occupational health and safety of farm staff [32,33]. Although there are no legal requirements regarding concentrations of ammonia in pig buildings, studies recommend that levels should be kept below 25 ppm, and ideally below 10 ppm [10,27]. In our study, maximum daily averages reached 22.5 ppm, with mean daily averages varying between 6–13 ppm. Overall, we recorded lower NH_3_ concentrations during summer, which is concordant with a trend reported by Chantziaras et al. [17]. Moreover, in agreement with several studies, NH_3_ concentrations were higher during the autumn/winter periods [17,34,35,36]. Interestingly, our cross-correlation analysis shows that higher NH_3_ concentrations were significantly associated with decreased ventilation rates, a link that was suggested but not statistically proven [17,36]. However, this correlation was less apparent during summer. It is possible that emptying of slurry tanks in the farm during this time of the year can explain this finding.

Furthermore, for the trial pigs reared during autumn/winter (3rd batch in room B) we also found that the RDI was best predicted by the exposure to lower relative humidity and higher ventilation rates with a lag of 7 and 15 days, respectively (adjusted R^2^ = 39%). As reviewed by Boyle et al. [37] there is strong evidence that high ventilation rates, i.e., draughts are risk factors for respiratory disease. Indeed, draughts are associated with increased frequencies of coughing and sneezing [38], and with the prevalence of pleurisy [39]. Conflicting with our results, high relative humidity is associated with respiratory disease [40], although most studies were carried out in farms where disease was present, meaning that this association may be an indirect one, as relative humidity facilitates dissemination of bacterial pathogens [6].

Regarding particulate matter, we recorded concentrations varying between 264 to 2924 µg/m^3^. These results are in line with the values reported in other studies carried out in finisher farms [10,41]. Furthermore, the majority of microorganisms are present in particles of size 10 µm [10], thus we restricted our measurements to PM_10_.

Considering the manual assessments carried out in this study, we only found a weak positive correlation between sneezing frequency and particulate matter. It is well established that dust irritates pigs’ respiratory epithelium [9], thus this association is coherent. However, we also found strong negative associations between particulate matter and ventilation flow, which is not in agreement with the literature [42]. Indeed, we recorded the lowest concentrations of PM during autumn/winter (when ventilation rates were lowest). Nevertheless, Wang et al. [43] suggest that as ventilation rates increase, PM is better mixed with air within an airspace, meaning that higher ventilation leads to a decrease of the overall mean particulate matter concentration. Still, the fact that we did not measure PM continuously is a limitation of this study.

Ultimately, our results suggest that coupling continuous environmental-oriented data and animal-oriented data may be useful to better understand pigs’ respiratory health, and as suggested by Chantziaras et al. [17] could help to elucidate the complexity of the Porcine Respiratory Disease Syndrome.

## 5. Conclusions

To our knowledge, this is the first study to investigate coughing in a farm where there were no pathogens associated with the PRDC. Results of this study can be used as guidelines on coughing levels in healthy pigs, and to calibrate the alarm systems of tools that measure coughing frequency, such as the cough monitor used in this study.

Furthermore, we show that environmental risk factors are to some extent associated with the respiratory health of pigs, thus we suggest that information collected on these risk factors should be used to help with decision-making processes on farm. We highlight the importance of continuously measuring ammonia concentrations, and urge for the integration of sensor technology and ventilation systems.

## Figures and Tables

**Table 1:** Descriptive statistics for three environmental parameters, ventilation flow, and the Respiratory Distress Index (RDI) summarized over the whole time period for each batch and room.

Replicate	[NH_3_] ^1^	Temperature °C	Relative Humidity %	Ventilation ^2^	RDI
First batch					
**Room A**					
Mean (sd)	11 (4.3)	21 (0.7)	65 (3.9)	43 (18.6)	1.6 (5.22)
Median (min–max)	10 (3.2–22.0)	21 (22.0–22.5)	65 (56.8–73.5)	39 (13.8–84.8)	0.2 (0–31.87)
**Room B**					
Mean (sd)	9 (3.1)	21 (0.7)	68 (3.8)	43 (13.9)	0.4 (0.31)
Median (min–max)	9 (2.7–15.7)	22 (20.0–22.6)	68 (58.6–74.3)	41 (20.9–75.5)	0.25 (0–1.5)
Second Batch					
**Room A**					
Mean (sd)	10 (2.6)	22 (0.6)	69 (4.1)	80 (14.0)	0.2 (0.15)
Median (min–max)	9 (2.7–15.9)	22 (20.3–23.6)	69 (61.8–78.1)	81 (28.5–103.0)	0.11 (0–0.6)
**Room B**					
Mean (sd)	6 (2.2)	22 (0.7)	72 (4.3)	87 (12.7)	0.3 (0.36)
Median (min–max)	6 (1.6–13.5)	22 (20.5–24.2)	71 (64.1–80.7)	87 (44.7–108.0)	0.21 (0–2.1)
Third Batch					
**Room A**					
Mean (sd)	13 (3.6)	20 (1.3)	70 (2.9)	39 (19.0)	0.3 (0.86)
Median (min–max)	14 (3.5–21.3)	19 (18.5–21.5)	70 (64.2–84.5)	37 (15.1–82.9)	0.11 (0–4.71)
**Room B**					
Mean (sd)	10 (3.3)	20 (1.6)	72 (3.1)	41 (18.9)	0.2 (0.30)
Median (min–max)	9 (3.9–22.5)	19 (18.5–22.4)	72 (66.4–88.7)	39 (15.9–81.2)	0.11 (0–1.99)

^1^ [NH_3_]: ammonia concentrations (ppm); ^2^ m^3^/h.

**Table 2:** Descriptive statistics for the concentration of Particulate Matter (PM_10_) and coughing and sneezing frequencies assessed manually.

Replicate	PM_10_ ^1^	Coughing Frequency ^2^	Sneezing Frequency ^3^
First batch (*n* = 33)			
**Room A**			
Mean (sd)	1346 (621.4)	0.0012 (0.00427)	0.005 (0.0050)
Median (min–max)	1230 (391.3–2924.3)	0 (0–0.021)	0.004 (0–0.0211)
**Room B**			
Mean (sd)	1435 (376.6)	0.0011 (0.00397)	0.004 (0.0032)
Median (min–max)	1405 (694.3–2039.7)	0 (0–0.021)	0.004 (0–0.0158)
Second batch (*n* = 32)			
**Room A**			
Mean (sd)	644 (186.2)	0.0001 (0.00044)	0.001 (0.0022)
Median (min–max)	623 (387.3–1061.0)	0 (0–0.002)	0 (0–0.0088)
**Room B**			
Mean (sd)	604 (154.7)	0.0007 (0.00176)	0.002 (0.0035)
Median (min–max)	589 (263.7–923.0)	0 (0–0.007)	0.002 (0–0.0126)
Third batch (*n* = 46)			
**Room A**			
Mean (sd)	1350 (447.0)	0.0005 (0.00097)	0.003 (0–0.0021)
Median (min–max)	1450 (441.0–2231.7)	0 (0–0.004)	0.002 (0–0.0087)
**Room B**			
Mean (sd)	1441 (370.4)	0.0012 (0.00181)	0.002 (0.0026)
Median (min–max)	1365 (763.7–2291.3)	0 (0–0.007)	0.002 (0–0.0139)

^1^ µg/m^3^; ^2^ Number of coughs/pig/min; ^3^ Number of sneezes/pig/min.

**Table 3:** Multivariable regression models of lagged environmental parameters from the Respiratory Distress Index.

Models	Predictors	Estimate (SE)	*p*-Value
Respiratory Distress Index (1st batch—room A) Adj. R^2^ = 47%	Intercept	−0.01 (0.426)	0.976
	[NH_3_] ^1^ lag −1	0.58 (0.184)	0.003 *
	[NH_3_] lag −7	0.70 (0.209)	0.001 *
	[NH_3_] lag −8	0.51 (0.210)	0.020
	[NH_3_] lag −15	0.98 (0.240)	<0.001 *
	RH ^2^ lag −3	−0.32 (0.127)	0.014
Respiratory Distress Index (3rd batch—room B) Adj. R^2^ = 39%	Intercept	−0.00 (0.033)	0.982
	RH lag −7	−0.07 (0.016)	<0.001 *
	RH lag −8	0.03 (0.016)	0.028
	Ventilation flow lag—15	0.01 (0.004)	0.003 *

^1^ Ammonia concentrations; ^2^ Relative humidity; * Indicates significant variables after Bonferroni corrections.

## Data Availability

The datasets used and analyzed during the current study are available from the corresponding author on reasonable request.

## Supplementary Materials

The following supporting information can be downloaded at: https://www.mdpi.com/article/10.3390/ani12080982/s1, Figure S1: Cross-correlation function for ammonia concentrations (A), temperature (B), relative humidity (C), and ventilation rate and the Respiratory Distress Index. Graphs (i–iii) correspond to the first, second, and third batches of room A, respectively. Graphs (iv–vi) correspond to the first, second, and third batches of room B, respectively. Blue lines indicate the significance threshold.
