# Supplementary material for: Environmental Risk Factors Influence the Frequency of Coughing and Sneezing Episodes in Finisher Pigs on a Farm Free of Respiratory Disease
Source: Animals (Basel). 2022 Apr 11;12(8):982. doi: 10.3390/ani12080982 (PMC9032133; doi:10.3390/ani12080982)
Supplement: Supplementary file 1 [file animals-12-00982-s001.zip › animals-1627777-supplementary-figure s1-caption.pdf]

A

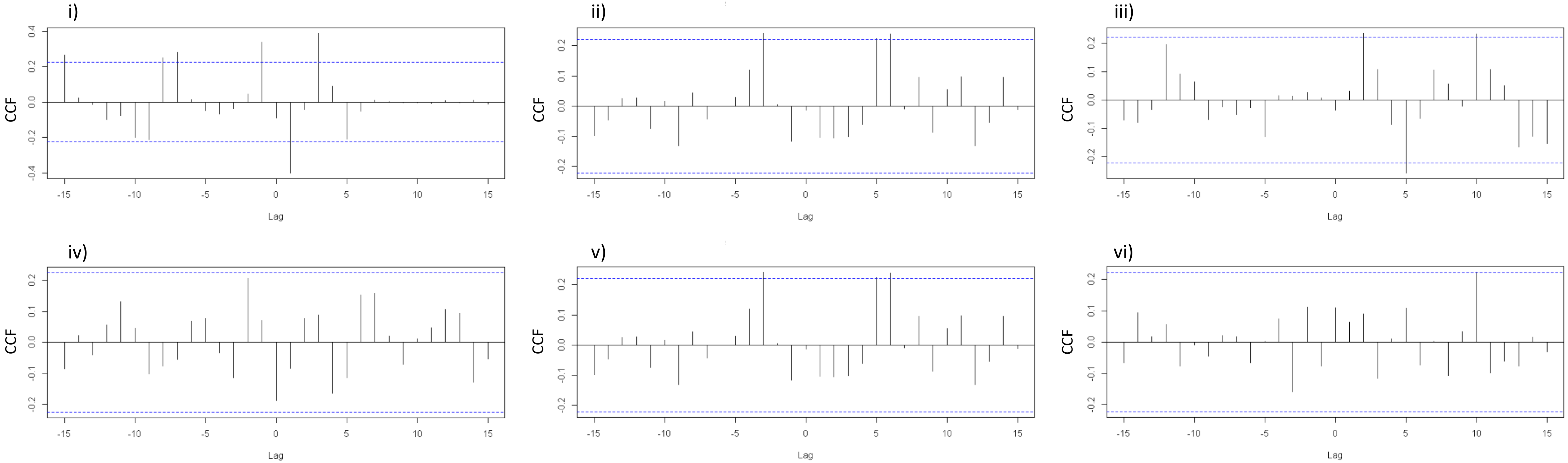

B

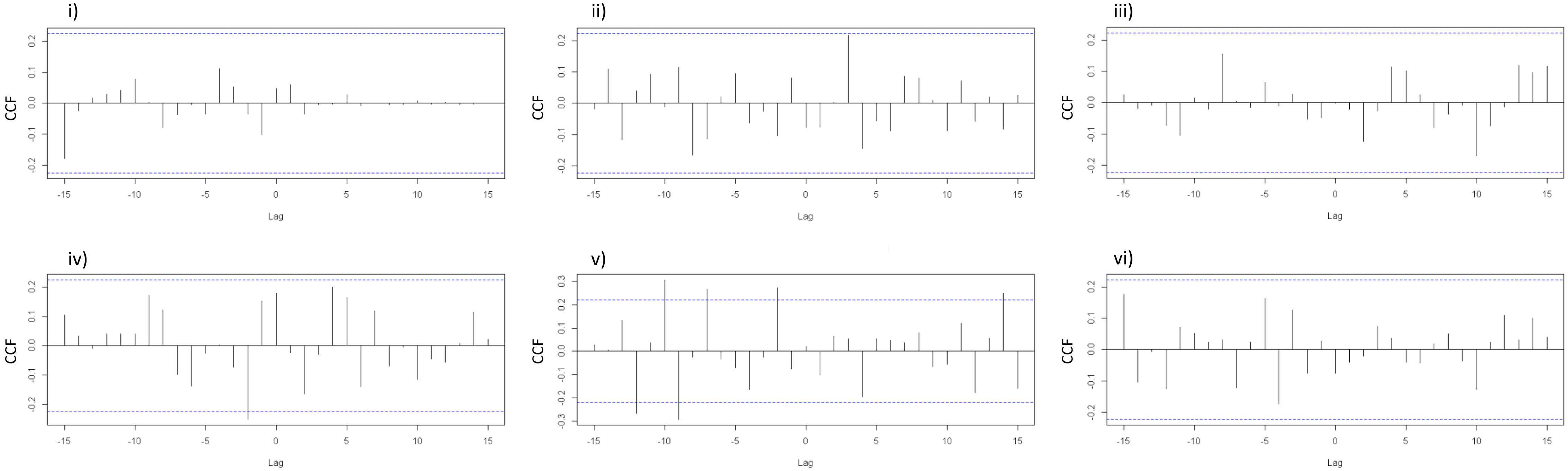

C

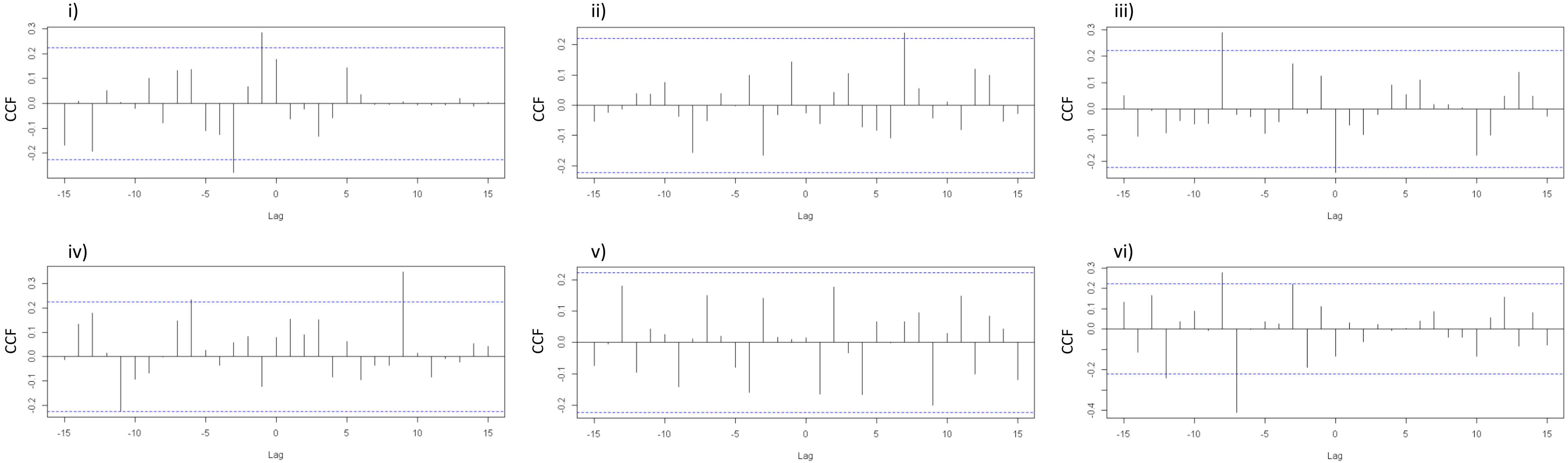

D

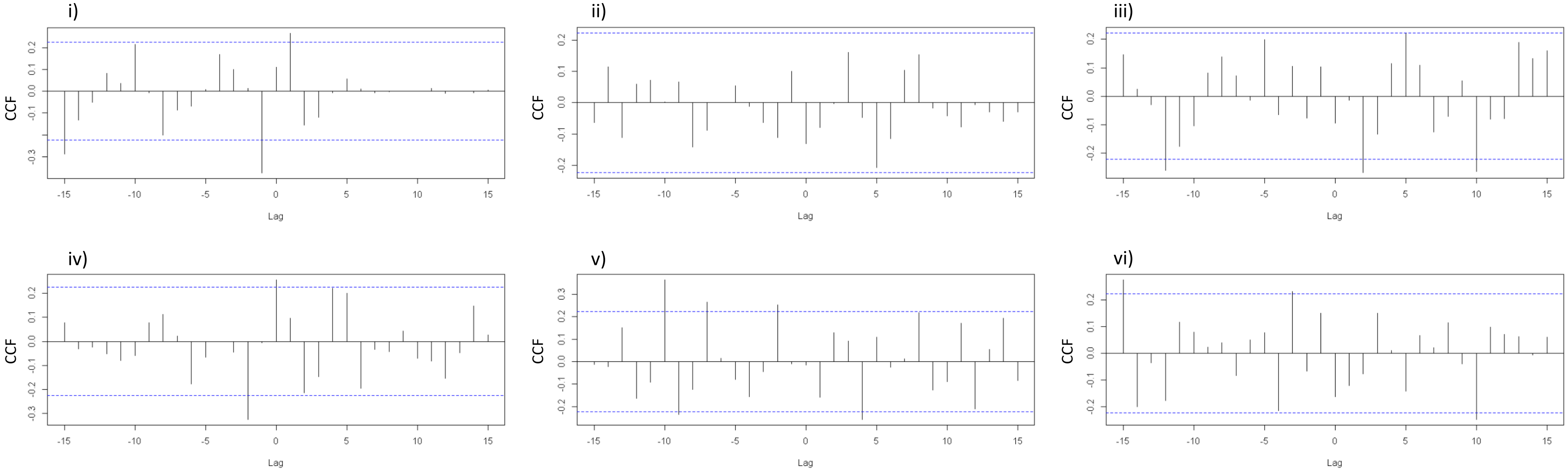

Figure S1: Cross-correlation function for ammonia concentrations (A), temperature (B), relative humidity (C), and ventilation rate (D) and the Respiratory Distress Index. Graphs (i–iii) correspond to the first, second, and third batches of room A, respectively. Graphs (iv–vi) correspond to the first, second, and third batches of room B, respectively. Blue lines indicate the significance threshold.
